# Histone crotonylation of peripheral blood mononuclear cells is a potential biomarker for diagnosis of colorectal cancer

**DOI:** 10.1186/s13072-023-00509-3

**Published:** 2023-09-26

**Authors:** Jia-Yi Hou, Ning Li, Jie Wang, Li-Juan Gao, Jia-Song Chang, Ji-Min Cao

**Affiliations:** 1 Department of Clinical Laboratory, Shanxi Provincial Academy of Traditional Chinese Medicine, Taiyuan, China; 2 Department of Gastrointestinal and Pancreatic Surgery and Hernia and Abdominal Surgery, Shanxi Provincial People’s Hospital, Taiyuan, China; 3 Key Laboratory of Cellular Physiology at Shanxi Medical University, Ministry of Education, Key Laboratory of Cellular Physiology of Shanxi Province, and the Department of Physiology, Shanxi Medical University, Taiyuan, China

**Keywords:** Colorectal cancer, Biomarker, Crotonylation, H2BK12, Peripheral blood mononuclear cells

## Abstract

**Background:**

Blood-based tests have public appeal in screening cancers due to their minimally invasive nature, ability to integrate with other routine blood tests, and high compliance. This study aimed to investigate whether certain epigenetic modulation of peripheral blood mononuclear cells (PBMCs) could be a biomarker of colorectal cancer (CRC).

**Results:**

Western blotting of histones in the PBMCs from 40 colorectal cancer patients and 40 healthy controls was performed to identify the crotonylation sites of proteins. The correlation of crotonylation with tumor staging and diagnostic efficacy were analyzed. Crotonylation of H2BK12 (H2BK12cr) was identified significantly upregulated in the PBMCs of CRC patients compared to healthy controls, and were closely related to distant metastasis (*P* = 0.0478) and late TNM stage (*P* = 0.0201). Receiver operator characteristic curve (ROC) analysis demonstrated that the area under curve (AUC) of H2BK12cr was 0.8488, the sensitivity was 70%, and the specificity was 92.5%. The H2BK12cr parameter significantly increased the diagnostic effectiveness of CRC compared with the commercial carcinoembryonic antigen assays.

**Conclusions:**

The H2BK12cr level in PBMCs of CRC patients has a potential to be a biomarker for distinguishing CRC patients from healthy controls with the advantages of easy operation and high diagnostic efficacy.

**Supplementary Information:**

The online version contains supplementary material available at 10.1186/s13072-023-00509-3.

## Introduction

Colorectal cancer (CRC) is one of the most common malignant tumors of the digestive system [1, 2]. Early diagnosis is the key measure to treat CRC patients earlier and improve the survival rate. About 90% of CRC patients diagnosed early can be cured by surgery. The median survival in untreated CRC liver metastases was only 6.9 months, and the 5-year survival in unresectable patients was less than 5% [3, 4]. Therefore, effective intervention after early diagnosis is of great significance to CRC patients. The diagnostic method of CRC is colonoscopy combined with pathological examination as the criteria, but because of the invasive nature of the measure, its compliance in the physical examination population is not high [5]. In current practice, noninvasive options will become increasingly important, especially utilization of new CRC biomarkers in the peripheral blood.

This study aimed to discover new biomarkers of CRC in the peripheral blood cells, especially the epigenetic markers in the leukocytes. It is known that nucleosomes are composed of four core histones (H2A, H2B, H3 and H4), which are the main components of chromatin. Histone is originally regarded as a static scaffold protein for DNA packaging, but now it has been proved to be a dynamic protein with many types of post-translational modifications, including acetylation, phosphorylation, methylation and ubiquitination [6, 7]. Lysine crotonylation (Kcr) on histones was first described in 2011 by Zhao and colleagues [8], and has been detected in the core histones of HeLa cells, mouse, cerevisiae, elegans, melanogaster, musculus, as well as plant [8–12]. Crotonylation was first identified as a specific marker of histone-associated gender-related genes, and has subsequently been found to play an important role in many diseases by regulating histone structure and function, such as acute kidney injury, depression, HIV latency, and cancer processes [13–16]. The study of crotonylation of histone protein in disease provides support for the development of biomarkers and therapeutics [17, 18].

It has been reported that the level of crotonylation is upregulated in colon cancer tissues compared to adjacent normal tissues [19]. However, peripheral blood testing is more acceptable than colonoscopy as a screening tool for CRC. Peripheral blood mononuclear cells (PBMCs) are peripheral blood cells with mononuclear nuclei, including lymphocytes and monocytes, mainly lymphocytes. Crotonylation of PBMCs has been shown associated with the progression of some diseases. Using tandem mass spectrometry and high-resolution liquid chromatography, Lin et al. [20] found crotonylation in a large number of proteins of PBMCs in patients with immunoglobulin A nephropathy (IgAN), which may be a diagnostic marker of IgAN. Nevertheless, whether crotonylation of PBMC is associated with CRC and can be used as an early diagnostic marker for CRC has not been proved.

In the present work, we used mass spectrometry-based modified proteomics to obtain a crotonylome data set of colorectal cancer and paracancer normal tissues. Bioinformatic analysis of proteins containing quantitative information sites revealed that some of the crotonylation-modified proteins were enriched in immune-related pathways. Recent studies suggest that immune cells circulate between peripheral blood and tumor tissues [21, 22]. Therefore, crotonylation of immune-related proteins in CRC tissues are increased, suggesting that crotonylation may be differentially expressed in peripheral blood immune cells of CRC patients. Here, we investigated the crotonylation of proteins in PBMCs from CRC patients and healthy controls. We discovered that the crotonylation of H2BK12 (H2BK12cr) was significantly increased in the PBMCs of CRC, and high H2BK12cr level was closely related to late TNM stage. In addition, we evaluated the diagnostic potential of H2BK12cr by ROC curve, and revealed the possibility of H2BK12cr as a diagnostic marker of CRC.

## Materials and methods

### Participants

Forty patients diagnosed as CRC and forty healthy controls from Shanxi Provincial Academy of Traditional Chinese Medicine (Taiyuan, China) were enrolled in the study. Those with other cancers or receiving any preoperative chemotherapy or radiotherapy were not eligible. Relevant clinical data were collected from patients’ clinical records. Demographics of patients and healthy volunteers are shown in Additional file 1: Table S1. Differences in gender between the two groups were analyzed by Fisher's exact test and the differences of height and weight were evaluated with a two-tailed unpaired Student’s *t* test. Differences in the distribution of the three age groups between CRC and healthy controls were tested by Chi-square. There were no statistical differences in gender, age, body weight and height between the two groups (*P* > 0.05). The research protocol was approved by the Ethic Committee of Shanxi Provincial Academy of Traditional Chinese Medicine (Approval No.: 2019-06KY005). The entire experimental protocol was conducted in compliance with the institutional guidelines. Four paired tissue samples from the patients were processed for proteome profiling with the technical support of Jingjie PTM BioLabs.

### Trypsin digestion and affinity enrichment of crotonylated peptides

For tissue protein digestion, dithiothreitol was added to the protein solution to reduce the final concentration of dithiothreitol to 5 mM for 30 min at 56 °C and alkylated with 11 mM iodoacetamide for 15 min at room temperature in darkness. Then, the urea concentration of the sample was diluted to less than 2 M. At the mass ratio of 1:50 (trypsin:protein), trypsin was added for the first digestion overnight at 37 °C and 1:100 (trypsin:protein) for a second 4 h digestion.

For pan antibody-based PTM enrichment, the peptides were dissolved in IP buffer solution and transferred to pre-washed crotonyl resin (PTM-503, PTM Bio) at 4 °C overnight with gentle shaking. After incubation, the beads were washed four times with IP buffer and twice with deionized H_2_O. Then, 0.1% trifluoroacetic acid elution solution was used to elute the peptides bound to the resin for three times, and the eluent was collected and dried by vacuum freezing. Finally, the resulting peptides were desalted with C18 ZipTips (Millipore) and analyzed by liquid chromatography–mass spectrometry (LC–MS).

### LC–MS/MS analysis

The tryptic peptides were dissolved in solvent A (0.1% formic acid in water) and loaded directly onto a home-made reversed-phase analysis column (25-cm length, 75 μm i.d.). On the nano-elution UHPLC system (Bruker Daltonics), polypeptides were graded with a gradient from 6% to 22% solvent B (0.1% formic acid in acetonitrile) over 43 min, 22–30% in 13 min and climbing to 80% in 2 min, and then maintained for the last 2 min at a rate of 80%, all at a constant flow rate of 400 nL/min.

The peptides were treated with capillary source and analyzed by timsTOF Pro (Bruker Daltonics) mass spectrometry. The timsTOF Pro was operated in parallel accumulation serial fragmentation (PASEF) mode.

### Database search and bioinformatics analysis

The resulting LC–MS/MS data were processed using Maxquant search engine (v.1.6.6.0). Tandem mass spectra were searched against the Human_SwissProt concatenated with reverse decoy database. Trypsin/P was specified as cleavage enzyme allowing up to 4 missing cleavages. The mass tolerance for precursor ions was set as 40 ppm in First search and 40 ppm in Main search, and the mass tolerance for fragment ions was set as 0.04 Da. Carbamidomethyl on cysteine was specified as fixed modification, and acetylation on protein N-terminal, oxidation on methionine, and crotonylation on lysine were specified as variable modifications. False discovery rate (FDR) was adjusted to < 1%. The criterium of fold-change selection was 2. Gene Ontology (GO) annotation proteome was derived from the UniProt-GOA database (V.5.14-53.0).

### Blood sampling and PBMCs isolation

A 10-mL peripheral whole blood sample was collected via vein puncture at 8:00 am from each participant into heparinized vacutainers. Plasma samples were obtained by centrifugation of the blood samples at 3000*g* for 15 min at room temperature, and three carcinoembryonic antigens in the plasma including CEA, CA199 and CA724 were detected by chemiluminescency (Dxl800 Access, BECKMAN COULTER). PBMCs were obtained via density gradient centrifugation at 1000*g* for 10 min at room temperature using Hypaque–Ficoll (GE Healthcare Life Sciences).

### Protein extraction

The collected PBMCs were transferred to a 5-mL centrifuge tube. Four volumes of RIPA buffer (Thermo Scientific, USA) and 1 mM PMSF were added, then the cells were sonicated on ice three times with a high-intensity ultrasound processor (Scientz). Cell debris was further centrifuged at 12000*g* and 4 °C for 10 min. Finally, the supernatant was collected and cell protein concentration was determined by a BCA kit.

### Isolation of histones

PBMCs isolated from the vein blood were pelleted by centrifugation at 1000 rpm for 5 min at 4 °C. Cells were resuspended in pre-lysis buffer at 10^7^ cells/mL. Cells were lysed on ice for 10 min with gentle stirring, then were centrifuged at 3000 rpm for 5 min at 4 °C. Cell pellets were resuspended in three volumes (approximately 200 µL/10^7^ cells) of lysis buffer and were incubated on ice for 30 min, followed by centrifugation at 12,000 rpm for 5 min at 4 °C. The supernatant fractions (containing acid-soluble proteins) were transferred to a new vial. Balance-DTT buffer was prepared by adding DTT solution to the balance buffer at a 1:500 ratio. About 0.3 volumes of the balance-DTT buffer were added to the supernatant immediately. Protein concentration was quantified with an OD reading. BSA was used as a standard. The extracts were aliquoted and stored at − 20 °C for several days or stored − 80 °C before experiments. Repeated thawing and freezing were avoided.

### Western blotting

Protein samples from PBMCs were homogenized in lysis buffer (50 mM Tris HCl, 150 mM NaCl, 2 mM EDTA, 2 mM EGTA, 0.2% Triton X-100, 0.3% NP-40, 0.1 mM PMSF and 1 µg/mL pepstatin A). Equal amounts of proteins (20 μg) were separated on 10% SDS–PAGE (Invitrogen, USA) gels, transferred onto PVDF membranes (Millipore, USA), and then blocked with 5% skim milk at room temperature for 2 h. The proteins were incubated with the following antibodies for overnight, washed with PBS, then were incubated with horseradish peroxidase-labeled secondary anti-mouse or anti-rabbit antibodies (1:2000) (ZSGB-Bio, Beijing, China) for 1 h at room temperature. Target signals were detected using the ECL detection kit (Solarbio, China). The intensities of blots were quantified by ImageJ software and relative protein levels were normalized by H3. Commercial antibodies used were as follows: anti-PanKcr (1:1000, #501), H2BK11cr (1:2000, #508), H2BK12cr (1:2000, #509), H2BK20cr (1:2000, #512) and H2BK34cr (1:2000, #514) from PTMbio, China, and anti-H3 (1:2000) from CST, USA.

### Statistical analysis

Statistical analyses were performed using SPSS 19.0 software and GraphPad Prism V7. Results were expressed as mean ± standard error of mean (SEM). Difference between two groups was evaluated with grouped *t* test, and multiple-group comparison was performed using a two-way ANOVA test. Categorical data were calculated by Fisher's exact test or Chi-square test. *P* value of < 0.05 was considered statistically significant. Receiver operating characteristic (ROC) curves for single proteins were analyzed using GraphPad Prism. The ROC curves were generated by plotting the sensitivity and specificity of data based on a series of cutoff values. Sensitivity referred to the rate of true positives (CRC patients) correctly identified. Specificity was the rate of true negatives (control subjects) correctly identified, and AUC values were calculated. An AUC value of 1.00 indicated that the test was 100% accurate and every positive or negative sample was correctly identified.

## Results

### Histone crotonylation is increased in peripheral blood mononuclear cells from colorectal cancer patients

**Fig. 1:**
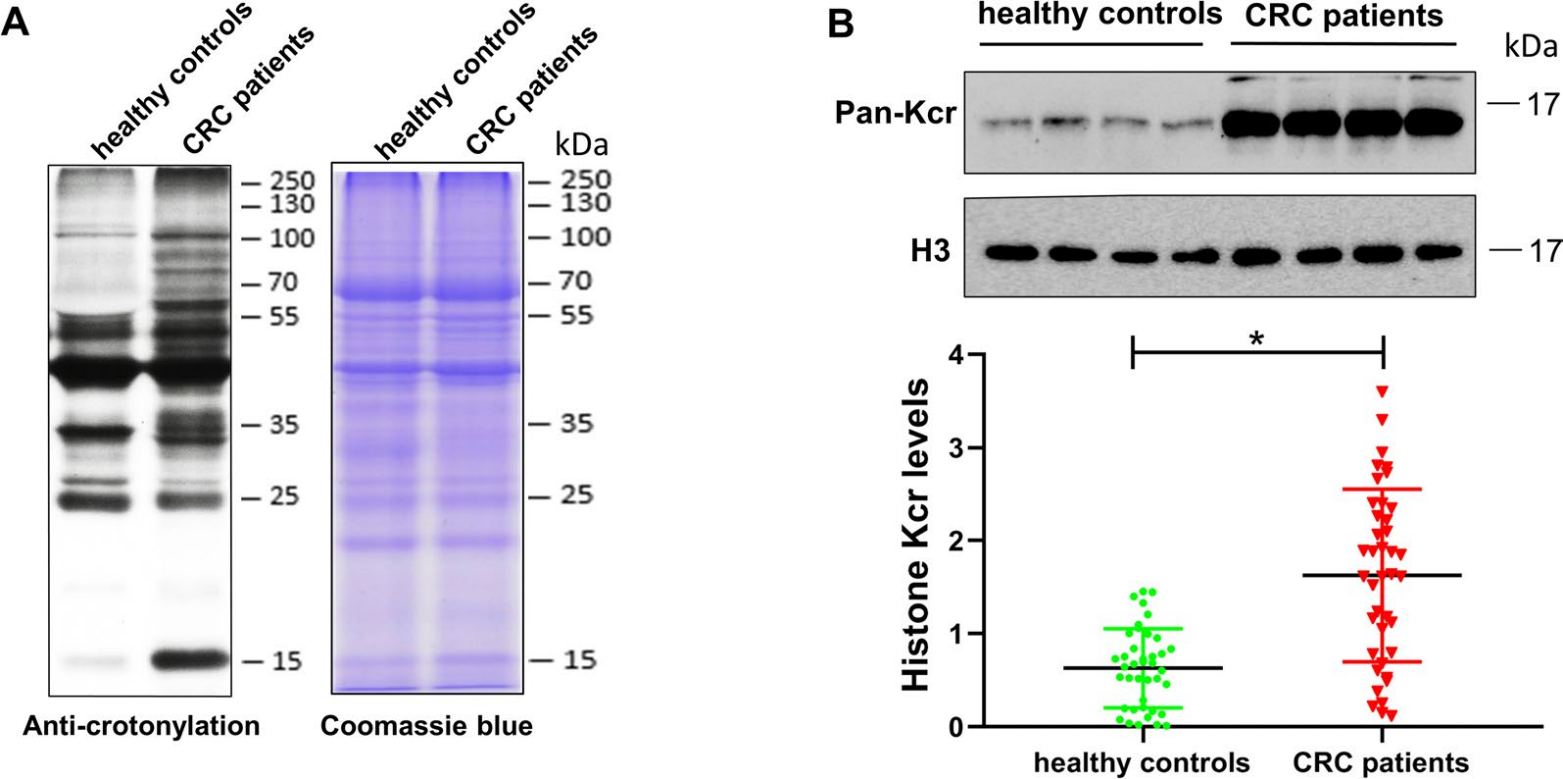
Elevation of histone crotonylation level in the peripheral blood mononuclear cells (PBMCs) of colorectal cancer (CRC) patients. **A** Identification of lysine crotonylation (Kcr) presented in the PBMCs of healthy controls and CRC patients using anti-crotonylation antibody. The protein loading amount in each lane was 20 µg extracted from the lysates of PBMCs. The SDS–PAGE gel stained with Coomassie brilliant blue showed the presence of proteins. **B** Western blots of histone crotonylation of the protein lysates of PBMCs from healthy controls and CRC patients (*n* = 40). Anti-histone H3 antibody was used as the loading control. Results were expressed as percentage change of crotonylated histones over control. The intensity of Kcr was quantified using ImageJ software, followed by statistical analysis. Each scale bar represents the mean ± SD of the 40 samples. The means were normalized to H3 (*P < 0.05 vs. healthy controls)

To detect the existence of crotonylated proteins in peripheral blood of CRC subjects, we first performed an immunoblotting analysis on the cell lysates of PBMCs from 40 CRC patients and 40 healthy controls using anti-PanKcr antibody. A representative pair of bands show multiple crotonylation-positive bands, indicating that crotonylation is a prevalent PTM in PBMCs (Fig. 1A). Cell separation into nuclei and cytosol disclosed a crotonylated protein band in the nuclei that corresponded in size to histones (Fig. 1A). Furthermore, histones were isolated from cell lysates and signal quantification of western blot disclosed an increase in overall histone crotonylation in the PBMCs of CRC patients (Fig. 1B). These results suggest that crotonylated histones are highly and differentially expressed in the PBMCs of CRC subjects compared to healthy controls.

### H2BK12 is the main site of increased crotonylation of histones

After using anti-PanKcr antibody to recognize all crotonylated lysine residues in histone proteins, it is necessary to identify the crotonylated proteins as well as their sites. In previous studies, this was often achieved by LC–MS/MS [23]. The integrated approach of SILAC labeling and affinity enrichment followed by LC–MS/MS can be utilized to identify the crotonylated core histone lysine sites and quantify the differential levels of histone Kcr which in turn corresponded with western blot results [24].

**Fig. 2:**
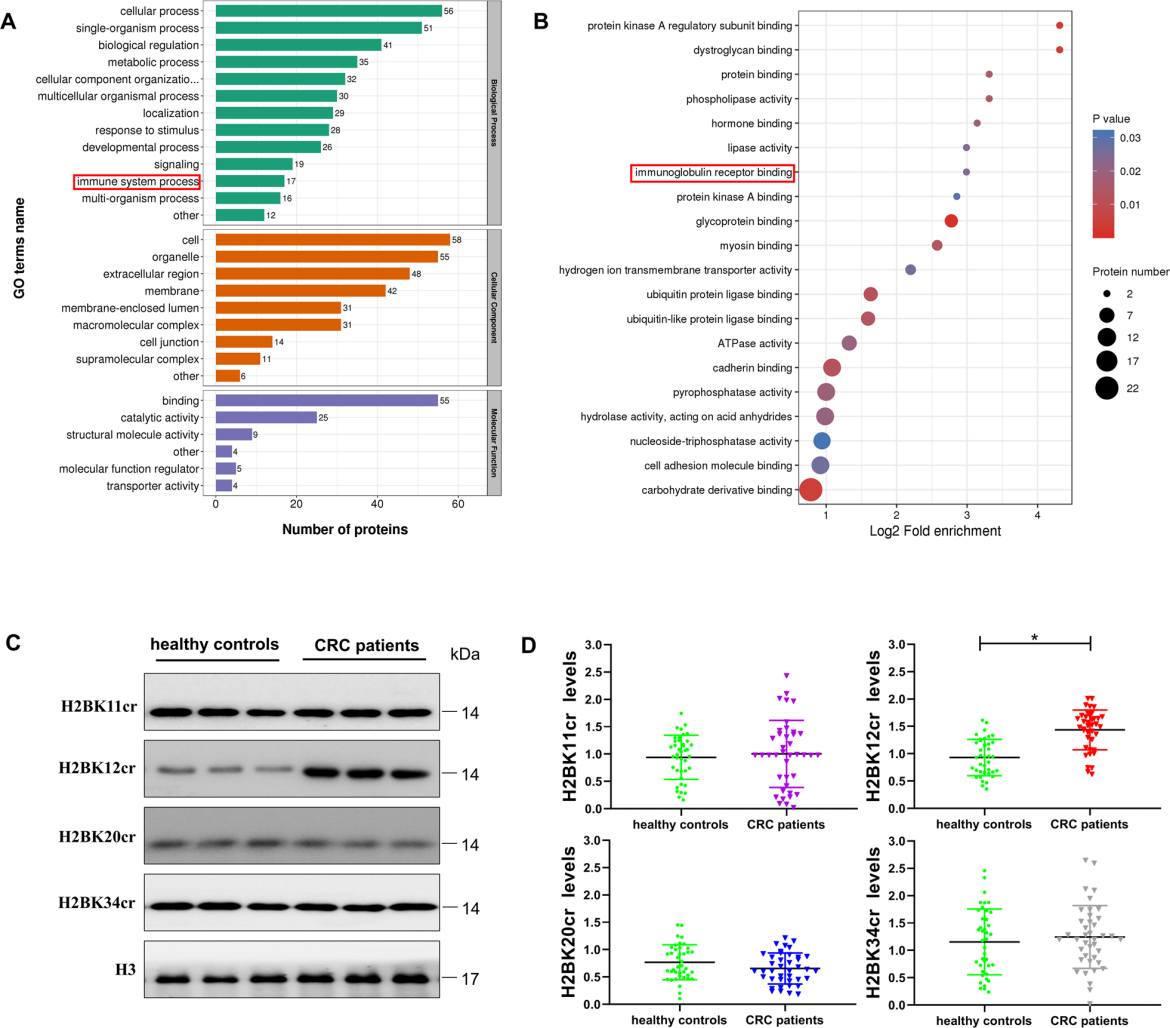
H2BK12 as the main site of elevated histone crotonylation. **A** Distribution of proteins corresponding to different crotonylation modification sites in GO secondary classification. **B** Bubble map of enrichment and distribution of proteins corresponding to different crotonylation modification sites in GO functional classification. **C** Crotonylation levels of H2BK11, H2BK12, H2BK20 and H2BK34 in CRC patients and healthy controls (*n* = 40) measured by western blotting using antibodies against the indicated histone crotonylations. Three representative blots were presented. **D** Quantification of relative histone H2B levels in the 40 pairs of samples tested. The intensity of crotonylation was quantified using the ImageJ software, followed by statistical analysis. Each scale bar represents mean ± SD for 40 samples. Mean data are normalized to H3 (*P < 0.05 vs. healthy controls)

We used LC–MS for crotonylomics analysis to quantify crotonylated proteins and their sites in four pairs of CRC tissues and peritumoral tissues. After normalizing the protein quantitative group to remove the effect of protein expression on the modification, we found that the modification levels of 65 protein sites were upregulated and 4 sites were downregulated compared with those in paracancerous tissues, including a subtype of histone H2B, namely, HIST1H2BL [25] (Table 1, Additional file 1: Table S2). GO classification showed that the upregulated proteins were involved in a variety of biological processes, including immune system process (Fig. 2A). The enrichment pathways of Cellular Components in GO classification were also concentrated in immune-related pathways, such as immunoglobulin receptor binding (Fig. 2B). Won et al. [22] found that circulating lymphocytes clustered into the tumor microenvironment in patients with mucosal-associated cancers, such as colon cancer, while Sundstrom et al. [21] reported that T lymphocytes were significantly accumulated in tumor tissues, irrespective of tumor stage or localization. Given that human immune cells circulate between peripheral blood and tumor tissue, we hypothesized that the elevated crotonylation level of H2B subtype in CRC tissues indicated similar changes in PBMCs.

**Table 1:** Difference of HIST1H2BL crotonylation between CRC and healthy subjects

Protein accession	Protein description	Gene name	Crotonylated amino acid	Regulated type	T/N ratio	T/N *P* value
Q99880	Histone H2B type 1	HIST1H2BL	K	Up	2.966	0.035123

To identify the sites of crotonylation in PBMCs, we detected the crotonylation levels at sites H2BK11, H2BK12, H2BK20 and H2BK34 of histones extracted from PBMCs proteins in 40 CRC patients and 40 healthy controls, respectively (Fig. 2C). We found that H2BK12cr expression levels were relatively elevated in CRC patients (Additional file 1: Fig. S1). Statistical results disclosed an increase in histone H2BK12 crotonylation in PBMCs proteins of CRC patients (Fig. 2D). These results reveal a potential role for high levels of crotonylation of H2BK12 in CRC patients.

### Crotonylation of H2BK12 correlates with distant metastasis and late TNM stage in CRC

**Fig. 3:**
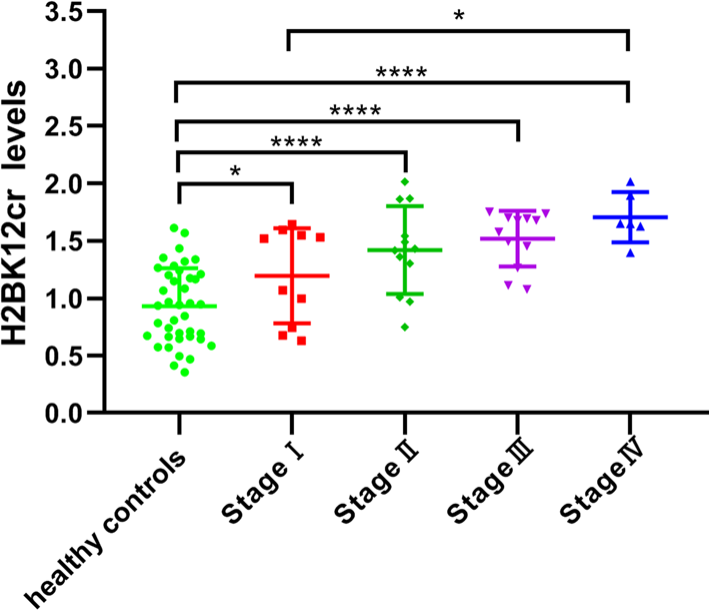
Association of H2BK12cr level with TNM stages of CRC. The 40 CRC patients were classified into TNM stages I to IV according to their pathological examination results. The H2BK12cr levels in PBMCs from 40 CRC patients and 40 healthy controls were analyzed. Statistical significance was assessed with a one-way analysis of variance (ANOVA) followed by Dunnett multiple comparisons test. Mean ± SD. *N* = 40 for healthy controls; *N* = 10, 12, 12 and 6 for stages I, II, III and IV, respectively. NS, not significant. *P < 0.05

To evaluate whether H2BK12cr levels were associated with the clinicopathological characteristics of CRC patients, we analyzed the H2BK12 crotonylation levels in PBMCs from 40 CRC patients and 40 healthy controls as described above. Results show that H2BK12cr levels in the PBMCs of CRC patients had no significant differences with regard to gender, age, tumor size and tumor differentiation degree, whereas were closely related to distant metastasis (*P* = 0.0478) and TNM stage (*P* = 0.0325) (Table 2). In addition, after classifying the samples according to the TNM stage of the patients, we found that H2BK12cr levels were elevated from stage I to stage IV compared with healthy controls, but H2BK12cr levels showed no significant difference among TNM stages I, II and III. Notably, there was a significant difference in the H2BK12cr level between TNM stage I and stage IV (*P* = 0.0277) (Fig. 3). These results suggest that the H2BK12cr level in PBMCs can distinguish healthy controls from CRC patients with stages I–IV, and the higher H2BK12cr level in CRC patients can predict the advanced progression.

**Table 2:** Association of H2BK12cr levels with clinicopathologic variables in CRC patients

Parameters	CRC patients (*n* = 40)	H2BK12cr level	*P* value
*Gender*			
Female	22	1.453 ± 0.2286	0.7418
Male	18	1.414 ± 0.4872	
*Age (year)*			
≤ 55	3	1.508 ± 0.4005	0.5151
56–65	31	1.459 ± 0.3549	
≥ 66	6	1.278 ± 0.416	
*Tumor size (cm)*			
< 2	16	1.243 ± 0.5045	0.1206
2–5	9	1.582 ± 0.2663	
> 5	18	1.400 ± 0.3124	
*Tumor differentiation*			
Well	12	1.39 ± 0.4225	0.2989
Moderate	15	1.391 ± 0.3268	
Poor	13	1.529 ± 0.3559	
*Distant metastasis*			
M0	34	1.388 ± 0.3651	0.0478^a^
M1	6	1.704 ± 0.2186	
*TNM stage*			
I	10	1.195 ± 0.4152	0.0325^a^
II	12	1.418 ± 0.3824	
III	12	1.316 ± 0.2428	
IV	6	1.704 ± 0.2186	

^a^Statistically significant (*P* < 0.05)

### The H2B12Kcr yields a better diagnostic performance than the commonly used carcinoembryonic antigens

Except for checking the H2B12Kcr levels of PBMCs in all the 40 CRC patients and 40 healthy controls, corresponding plasma were also separated from the blood samples of all these subjects, and were used to examine the carcinoembryonic antigen (CEA) commonly used as diagnostic factors for digestive tract tumors, including CEA, CA199 and CA724, using a chemiluminescence method.

**Fig. 4:**
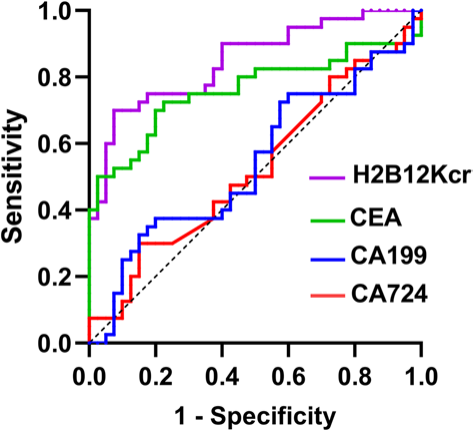
Receiver operator characteristic (ROC) analyses of H2B12Kcr, CEA, CA199 and CA724. ROC curve was created by plotting the true positive rate (sensitivity) against the false-positive rate (1-specificity)

To determine the sensitivity and specificity of H2B12Kcr, CEA, CA199 and CA724 in distinguishing CRC patients from healthy controls at diagnosis, ROC curves were generated (Fig. 4), and the area under curve (AUC) was calculated. Remarkably, H2B12Kcr had higher AUC (0.8488) with a 95% confidence interval (CI) of 0.7648 to 0.9327 (*P* < 0.001) (Table 3). The optimal thresholds of H2B12Kcr as a potential biomarker were determined by Youden’s index, and the sensitivity and specificity were, respectively, 70% and 92.5%, in CRC patients (Table 4). Compared with CEA, CA199 and CA724, H2B12Kcr seems to have better sensitivity and specificity in distinguishing CRC patients from healthy subjects.

**Table 3:** Area under curve (AUC) values of different diagnostic factors

Diagnostic factors	AUC	Standard error	95% confidence interval
H2B12Kcr	0.8488	0.04282	0.7648–0.9327
CEA	0.7606	0.05692	0.6491–0.8722
CA199	0.5259	0.06507	0.3984–0.6535
CA724	0.5425	0.06549	0.4141–0.6709

**Table 4:** Efficacy of H2B12Kcr as a potential diagnostic factor

Variable	Cut off value	Sensitivity	Specificity	Max Youden's index
H2B12Kcr	1.357	0.7	0.925	0.625

## Discussion

Colonoscopy is highly sensitive and specific for colorectal cancer detection and polyp removal, but it is invasive, expensive, and resource-intensive. Thus, although the slow growth of CRC provides a time window for early screening and avoiding threatened lesions, compliance with CRC screening remains low in many countries [26, 27]. Especially during the height of COVID-19 pandemic, CRC screening activities and colonoscopy procedures among patients decreased by 85–95% [28, 29]. There are approximately 10,000 deaths from breast cancer and CRC in the United States alone due to disruptions in pandemic-related care, and an estimated 18,800 people in the United States may experience a delay in CRC diagnosis [30]. A study reported that screening interruptions at up to 12 months could lead to a relative increase of 0.6–1.8% in CRC incidence, while providing immediate catch-up screening could reduce the impact of interruptions to less than 0.1% by the year of 2050 [31]. To improve current CRC screening rates and meet the need for multiple-modality CRC screening, many studies focus on the performance, risks and benefits of non-invasive screening tests. In the present study, we found that the H2B12Kcr level in PBMCs was significantly elevated in CRC patients. This approach using peripheral venous blood samples for CRC screening might become a new diagnostic strategy for CRC.

The possibility of using PBMCs as a CRC marker has been tested in recent years. Some lymphocytes among the PBMCs, such as CD4 + CD25 + Foxp3 + regulatory T cells (Tregs) and monocytic myeloid-derived suppressor cells (MMDSCs), have been found to be diagnostic and prognostic indicators of CRC [32, 33]. Ciarloni et al. [34] identified a 29-gene panel expressed in PBMCs that can be used for developing a novel minimally invasive test for accurate detection of adenomatous polyps and CRC using a standard real-time PCR platform. Shaath et al. [35] analyzed the transcriptome of PBMCs in CRC patients and healthy controls, and found that differently expression transcripts may be used as CRC biomarkers. Here, we further demonstrated that histone crotonylation of PBMCs at a specific site (H2BK12cr) is an alternative potential biomarker of CRC.

H2B is one of the earliest identified crotonylated histones, and H2BK12cr is a key link in the pathophysiological processes of some diseases. H2BK12cr can participate in the regulations of gene transcription, metabolism, DNA repair, depression, reproductive development and cardiac homeostasis [24]. Toxoplasma infection can inhibit H2BK12cr, which helps to inhibit epigenetic regulation and activate signaling pathways related to anti-infective immunity [23]. However, the role of H2BK12cr in tumors such as CRC is unclear.

Here, we found that H2BK12cr was upregulated in the PBMCs from CRC patients of stages I–IV compared with healthy individuals. We further investigated the clinical diagnostic potential of H2BK12cr using ROC curves and found that H2BK12cr had a sensitivity of 70% and a specificity of 92.5% in distinguishing CRC patients from healthy subjects (Table 4). Because the sample size was relatively smaller, the calculated specificity and sensitivity of H2BK12cr might be overestimated. However, the testing performance of H2BK12cr over CEA was still statistically significant. In CRC patients, higher levels of H2BK12cr were associated with poor prognosis, such as metastasis and advanced TNM stage. This finding suggests that H2BK12cr may have guiding implications for drug therapy and prognostic judgment of CRC. For example, monitoring the change of H2BK12cr during treatment may help to identify the progressive state of CRC.

Nevertheless, there is a limitation in the multi-approach validation of H2BK12cr in the study. The western blot results were not validated using an orthogonal methodology such as LC–MS/MS which might otherwise reduce the possibility of errors caused by the non-specific binding of antibodies. However, the application of LC–MS to detect PBMC requires the transfer of protein peptides to pre-washed crotonyl resin, which needs a large amount of protein. This is difficult for PBMC, especially when it is applied to clinical patients. In view of the circulation between PBMC and extracellular matrix of tumor, mass spectrometry was performed on tissues, where a large amount of protein was easily obtained, and the conclusion of H2BKcr elevation was also indirectly obtained in tumor patients.

There are multiple blood-based CRC screening tests in various stages of development. These tests range from CRC-specific tests to multi-cancer early detection tests. Candidate analytical targets include cell-free DNA (cfDNA), methylated circulating tumour DNA (ctDNA), gene-specific methylated septin 9 (mSEPT9), and combinations of methylated DNA and proteins [36–38]. The US Centers for Medicare and Medicaid Services (CMS) provide an approval threshold for a CRC blood test, i.e., a blood test needs to be 90% specific and 74% sensitive compared to an accepted standard, such as colonoscopy, and must be approved by the FDA and endorsed by at least one professional association guideline (CAG-00454N) in 2021. mSEPT9 is currently the only blood-based CRC test approved by the FDA for CRC screening in individuals who decline or are unable to complete the higher efficacy screening tests [39]. Its improved version has an overall sensitivity of 68% for CRC, which is close to our assay, but the sensitivity is only 64% for stages I–III disease [40]. Due to lack of sensitivity, it is still not approved for reimbursement by CMS [41]. Therefore, our currently proposed blood-based CRC detection, especially the H2BK12cr of PBMCs, still needs to be continuously improved in future studies before being used in the clinic in a standardized way. Alternatively, we may further explore the possibility of combining multiple indicators in diagnosis.

In fact, in underdeveloped areas where CRC blood screening methods are in urgent need of promotion, many laboratories cannot conveniently carry out PCR and other technologies, let alone omics technology. In the present study, the detection of H2BK12cr only requires the simple separation of PMBCs by centrifugation, western blotting and other basic means, which is an economical and simple method. Therefore, the use of H2BK12cr as a potential biomarker for CRC is attractive for most parts of the world. However, if H2BK12cr is applied to clinic, there may be inconsistency between batches and between laboratories due to method variability in PBMCs isolation, protein extraction and western blots. Nonetheless, according to the present study, a much high level of H2BK12cr in CRC identified by western blotting could still be an indicator/predictor of worse TNM stage and bad prognosis.

This exploratory work proposes the potentiality of H2BK12cr of PBMCs as a biomarker of CRC. This measure may have a good application prospect. There are still some limitations in the study. First, the study was conducted only in Han Chinese subjects, and the population size investigated was relatively small (only 40 colorectal cancer cases and 40 healthy controls). Further studies in larger and more diverse populations at multiple sites are needed to determine the effectiveness of H2BK12cr as a universal diagnostic tool. Second, this study only focused on the differential expression of H2BK12cr between CRC patients and healthy subjects, the potential significance of H2BK12cr in the early detection of other cancers still needs further exploration.

### Supplementary Information

**Additional file 1**: **Table S1.** Demographics of CRC patients vs. Healthy controls. **Table S2.** Differentially crotonylated proteins of CRC patients and healthy controls. **Fig S1.** H2BK12 is the main site of increased crotonylation of histones. The crotonylation levels of H2BK12 in CRC patients and healthy controls (n = 40) measured by western blotting. Anti-histone H3 antibody was used as the loading control. C: CRC patients; H: healthy controls.

## Data Availability

The data sets generated during and/or analyzed during the current study are available in the ProteomeXchange Consortium via the PRIDE [42] partner repository with the data set identifier PXD025709. Reviewer account details: Username: reviewer_pxd025709@ebi.ac.uk. Password: s5ucyJ3z.

## Abbreviations

AUC: Area under the curve
CEA: Carcinoembryonic antigen
cfDNA: Cell-free DNA
CI: Confidence interval
CMS: The US Centers for Medicare and Medicaid Services
CRC: Colorectal cancer
ctDNA: Circulating tumor DNA
H2BK12cr: Crotonylation of H2BK12
IgAN: Immunoglobulin A nephropathy
LC–MS: Liquid chromatography–mass spectrometry
Kcr: Lysine crotonylation
MMDSCs: Monocytic myeloid-derived suppressor cells
mSEPT9: Methylated septin 9
PBMCs: Peripheral blood mononuclear cells
Tregs: Regulatory T cells
