# Supplementary material for: Histone crotonylation of peripheral blood mononuclear cells is a potential biomarker for diagnosis of colorectal cancer
Source: Epigenetics Chromatin. 2023 Sep 26;16:35. doi: 10.1186/s13072-023-00509-3 (PMC10521402; doi:10.1186/s13072-023-00509-3)
Supplement: Supplementary file 1 — Additional file 1: Table S1. Demographics of CRC patients vs. Healthy controls. Table S2. Differentially crotonylated proteins of CRC patients and healthy controls. Fig S1. H2BK12 is the main site of increased crotonylation of histones. The crotonylation levels of H2BK12 in CRC patients and healthy controls (n = 40) measured by western blotting. Anti-histone H3 antibody was used as the loading control. C: CRC patients; H: healthy controls. [file 13072_2023_509_MOESM1_ESM.docx]

Supplementary Material

Histone crotonylation of peripheral blood mononuclear cells is a potential biomarker for diagnosis of colorectal cancer

Jia-Yi Hou^1^, Ning Li^2^, Jie Wang^3^, Li-Juan Gao^3^, Jia-Song Chang^3^, Ji-Min Cao^3,*^

^1^ Department of Clinical Laboratory, Shanxi Provincial Academy of Traditional Chinese Medicine, Taiyuan, China

^2^ Department of Gastrointestinal and Pancreatic Surgery & Hernia and Abdominal Surgery, Shanxi Provincial People’s Hospital, Taiyuan, China

^3^ Key Laboratory of Cellular Physiology at Shanxi Medical University, Ministry of Education, Key Laboratory of Cellular Physiology of Shanxi Province, and the Department of Physiology, Shanxi Medical University, Taiyuan, China

* Corresponding author. Email: [caojimin@sxmu.edu.cn](mailto:caojimin@sxmu.edu.cn)


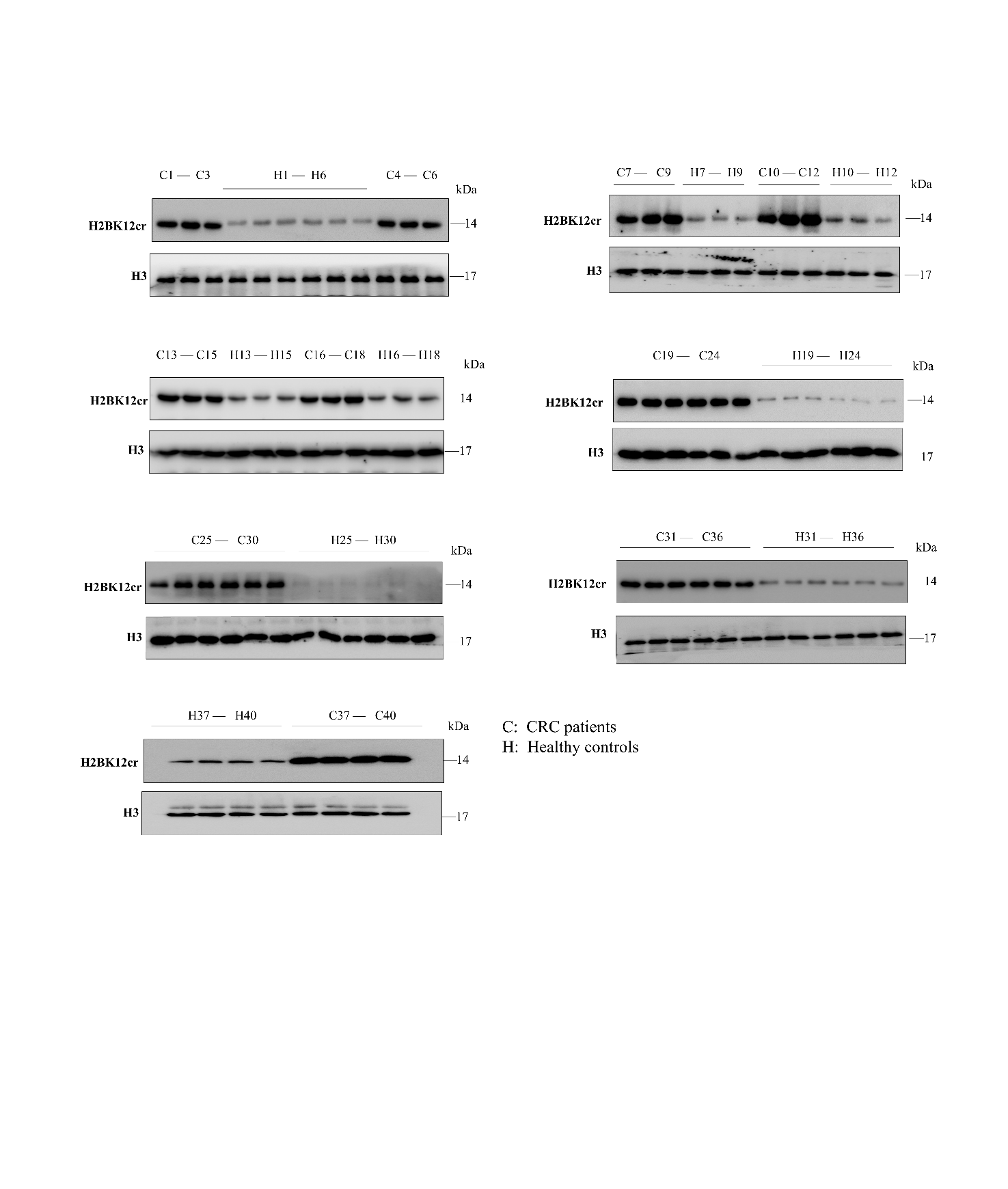


Figure S1. H2BK12 is the main site of increased crotonylation of histones.

The crotonylation levels of H2BK12 in CRC patients and healthy controls (n = 40) measured by western blotting. Anti-histone H3 antibody was used as the loading control. C: CRC patients; H: healthy controls.

Table S1. Demographics of CRC patients vs. Healthy controls

| Groups | Gender | | | Age | | | | | | Height | | Weight | |
| --- | --- | --- | --- | --- | --- | --- | --- | --- | --- | --- | --- | --- | --- |
|  | Male | Female | *P*-value | ≤55 | | 56-65 | | ≥66 | *P*-value | Mean±SD | *P*-value | Mean±SD | *P*-value |
| CRC patients | 18 | 22 | 0.2629 | 3 | 31 | | 6 | | 0.561 | 163.6±5.267 | 0.0788 | 70.8±9.985 | 0.0812 |
| Health controls | 24 | 16 |  | 6 | 29 | | 5 | |  | 166.1±7.365 |  | 67.13±8.570 |  |

CRC patients vs. Healthy controls  *P*>0.05

Table S2. Differentially crotonylated proteins of CRC patients and healthy controls

| Protein accession | Gene name | Amino acid | Regulated Type | T/N Ratio | T/N P value |
| --- | --- | --- | --- | --- | --- |
| O00499 | BIN1 | K | Up | 2.716 | 0.00173669 |
| O00560 | SDCBP | K | Up | 3.355 | 0.0093207 |
| O14979 | HNRNPDL | K | Up | 2.001 | 0.00180096 |
| O43776 | NARS | K | Up | 3.596 | 0.021599 |
| O75964 | ATP5MG | K | Up | 2.198 | 0.011678 |
| O95139 | NDUFB6 | K | Up | 2.012 | 0.049582 |
| O95994 | AGR2 | K | Up | 2.193 | 0.027064 |
| P00352 | ALDH1A1 | K | Up | 2.527 | 0.0181411 |
| P01591 | JCHAIN | K | Up | 2.496 | 0.0101798 |
| P02671 | FGA | K | Up | 2.009 | 0.0078838 |
| P04843 | RPN1 | K | Up | 2.481 | 0.0033213 |
| P06744 | GPI | K | Up | 2.077 | 0.0176818 |
| P07384 | CAPN1 | K | Up | 2.17 | 0.040661 |
| P08133 | ANXA6 | K | Up | 2.145 | 0.00118135 |
| P10809 | HSPD1 | K | Up | 2.198 | 0.010625 |
| P11142 | HSPA8 | K | Up | 2.058 | 0.0058218 |
| P11310 | ACADM | K | Up | 2.287 | 0.0079834 |
| P11532 | DMD | K | Up | 2.253 | 0.040024 |
| P12111 | COL6A3 | K | Up | 9.183 | 0.0049563 |
| P12268 | IMPDH2 | K | Up | 2.438 | 0.0253 |
| P12532 | CKMT1A | K | Up | 2.292 | 0.0142815 |
| P12956 | XRCC6 | K | Up | 8.351 | 0.024483 |
| P14555 | PLA2G2A | K | Up | 2.123 | 0.038201 |
| P15311 | EZR | K | Up | 2.041 | 0.0110787 |
| P16152 | CBR1 | K | Up | 2.002 | 0.020301 |
| P16949 | STMN1 | K | Up | 2.253 | 0.047456 |
| P17612 | PRKACA | K | Up | 2.931 | 0.0073614 |
| P20810 | CAST | K | Up | 4.958 | 0.0023608 |
| P21333 | FLNA | K | Up | 2.59 | 0.033618 |
| P26599 | PTBP1 | K | Up | 2.402 | 0.00072487 |
| P30050 | RPL12 | K | Up | 2.347 | 0.025864 |
| P30101 | PDIA3 | K | Up | 2.027 | 0.00090217 |
| P32456 | GBP2 | K | Up | 2.001 | 0.029301 |
| P35749 | MYH11 | K | Up | 2.573 | 0.030441 |
| P36542 | ATP5F1C | K | Up | 2.482 | 0.032401 |
| P36542 | ATP5F1C | K | Up | 2.302 | 0.037216 |
| P36543 | ATP6V1E1 | K | Up | 2.13 | 0.0026012 |
| P38646 | HSPA9 | K | Up | 2.082 | 0.0163354 |
| P50479 | PDLIM4 | K | Up | 2.024 | 0.0192758 |
| P51888 | PRELP | K | Up | 2.38 | 0.0105581 |
| P54652 | HSPA2 | K | Up | 2.074 | 0.021182 |
| P54652 | HSPA2 | K | Up | 5.029 | 0.045997 |
| P54652 | HSPA2 | K | Up | 3.462 | 0.0053432 |
| P61088 | UBE2N | K | Up | 2.331 | 0.00060089 |
| P61247 | RPS3A | K | Up | 2.151 | 0.025918 |
| P61769 | B2M | K | Up | 2.021 | 0.035341 |
| P62826 | RAN | K | Up | 2.529 | 0.032578 |
| P62913 | RPL11 | K | Up | 2.412 | 0.0117809 |
| Q05682 | CALD1 | K | Up | 2.489 | 0.0086036 |
| Q09666 | AHNAK | K | Up | 2.236 | 0.0025362 |
| Q09666 | AHNAK | K | Up | 2.347 | 0.0176179 |
| Q09666 | AHNAK | K | Up | 2.269 | 0.0141751 |
| Q14204 | DYNC1H1 | K | Up | 2.27 | 0.022358 |
| Q14247 | CTTN | K | Up | 2.071 | 0.0171001 |
| Q15181 | PPA1 | K | Up | 2.307 | 0.0146159 |
| Q16352 | INA | K | Up | 2.055 | 0.0038028 |
| Q86TX2 | ACOT1 | K | Up | 2.004 | 0.0157983 |
| Q8NC51 | SERBP1 | K | Up | 2.012 | 0.023665 |
| Q969G5 | CAVIN3 | K | Up | 2.839 | 0.021478 |
| Q99729 | HNRNPAB | K | Up | 3.341 | 0.0079035 |
| Q99880 | HIST1H2BL | K | Up | 2.966 | 0.035123 |
| Q9BPW8 | NIPSNAP1 | K | Up | 2.079 | 0.0164221 |
| Q9BTT6 | LRRC1 | K | Up | 2.001 | 0.033401 |
| Q9BXN1 | ASPN | K | Up | 2.179 | 0.0104776 |
| Q9H4M9 | EHD1 | K | Up | 2.399 | 0.034005 |
| P0DP25 | CALM3 | K | Down | 0.236 | 0.024142 |
| P51665 | PSMD7 | K | Down | 0.461 | 0.03028 |
| P68032 | ACTC1 | K | Down | 0.378 | 0.032902 |
| Q13895 | BYSL | K | Down | 0.459 | 0.0190601 |
